# Chromosomal genome assembly of the ethanol production strain CBS 11270 indicates a highly dynamic genome structure in the yeast species *Brettanomyces bruxellensis*

**DOI:** 10.1371/journal.pone.0215077

**Published:** 2019-05-01

**Authors:** Ievgeniia A. Tiukova, Mats E. Pettersson, Marc P. Hoeppner, Remi-Andre Olsen, Max Käller, Jens Nielsen, Jacques Dainat, Henrik Lantz, Jonas Söderberg, Volkmar Passoth

**Affiliations:** 1 Chalmers University of Technology, Department of Biology and Biological Engineering, Systems and Synthetic Biology, Göteborg, Sweden; 2 Swedish University of Agricultural Sciences, Department of Molecular Sciences, Uppsala, Sweden; 3 Uppsala University, Department of Medical Biochemistry and Microbiology, Uppsala, Sweden; 4 National Bioinformatics Infrastructure Sweden (NBIS), Uppsala, Sweden; 5 Christian-Albrechts-University of Kiel, Institute of Clinical Molecular Biology, Kiel, Germany; 6 Science for Life Laboratory, Division of Gene Technology, School of Biotechnology, Royal Institute of Technology (KTH), Solna, Sweden; 7 Royal Institute of Technology, Biotechnology and Health, School of Engineering Sciences in Chemistry, SciLifeLab, Stockholm, Sweden; 8 Stockholm University, Department of Biochemistry and Biophysics, SciLifeLab, Stockholm, Sweden; 9 Uppsala University, Department of Cell and Molecular Biology, Molecular Evolution, Uppsala, Sweden; University of Strasbourg, FRANCE

## Abstract

Here, we present the genome of the industrial ethanol production strain *Brettanomyces bruxellensis* CBS 11270. The nuclear genome was found to be diploid, containing four chromosomes with sizes of ranging from 2.2 to 4.0 Mbp. A 75 Kbp mitochondrial genome was also identified. Comparing the homologous chromosomes, we detected that 0.32% of nucleotides were polymorphic, i.e. formed single nucleotide polymorphisms (SNPs), 40.6% of them were found in coding regions (i.e. 0.13% of all nucleotides formed SNPs and were in coding regions). In addition, 8,538 indels were found. The total number of protein coding genes was 4897, of them, 4,284 were annotated on chromosomes; and the mitochondrial genome contained 18 protein coding genes. Additionally, 595 genes, which were annotated, were on contigs not associated with chromosomes. A number of genes was duplicated, most of them as tandem repeats, including a six-gene cluster located on chromosome 3. There were also examples of interchromosomal gene duplications, including a duplication of a six-gene cluster, which was found on both chromosomes 1 and 4. Gene copy number analysis suggested loss of heterozygosity for 372 genes. This may reflect adaptation to relatively harsh but constant conditions of continuous fermentation. Analysis of gene topology showed that most of these losses occurred in clusters of more than one gene, the largest cluster comprising 33 genes. Comparative analysis against the wine isolate CBS 2499 revealed 88,534 SNPs and 8,133 indels. Moreover, when the scaffolds of the CBS 2499 genome assembly were aligned against the chromosomes of CBS 11270, many of them aligned completely, some have chunks aligned to different chromosomes, and some were in fact rearranged. Our findings indicate a highly dynamic genome within the species *B*. *bruxellensis* and a tendency towards reduction of gene number in long-term continuous cultivation.

## Introduction

The yeast, *Brettanomyces bruxellensis* (syn. *Dekkera bruxellensis*- the last issue of the taxonomic monography of the yeasts [1] mentioned *D*. *bruxellensis* as the valid name of this species, however, according to the recently introduced principle “one species, one name” [2] we use the older name *B*. *bruxellensis* in this study), is regarded as a major contaminant in wine [3, 4] and bioethanol production [5, 6]. However, it is also involved in certain economically relevant, spontaneous fermentations, such as the production of Belgian Lambic beer [7–9]. It has also been found to be the production yeast in a continuous ethanol production process with cell recirculation, after outcompeting the initially inoculated *Saccharomyces cerevisiae* [10]. *B*. *bruxellensis* has an ethanol tolerance similar to *S*. *cerevisiae*, and has the ability to grow at low sugar concentrations. This explains why it usually becomes important in the later stages of wine or beer production, or in sugar limited continuous fermentations [11]. The mechanism of outcompeting *S*. *cerevisiae* is not completely known at present. It has been speculated that the ability of *B*. *bruxellensis* to assimilate nitrate may play a role, such as in some Brazilian ethanol production plants, where nitrate can come into the fermentation with the substrate, sucrose from sugarcane [12]. However, outcompetition of *S*. *cerevisiae* by *B*. *bruxellensis* has been observed in nitrate-free, glucose-limited fermentations, and thus, the competitiveness of the yeast could rather be due to a higher affinity for the substrate and/or a more efficient energy metabolism [13].

*B*. *bruxellensis* has several interesting metabolic capabilities, such as the (strain dependent) ability to ferment cellobiose to ethanol [14, 15], to assimilate nitrate [12] and even xylose [16]. Due to its robustness and its ability to assimilate the above-mentioned sugars, it has been regarded as a potential candidate to convert lignocellulose-hydrolysate to ethanol, and after some adaptation to the substrate, it performed as well as *S*. *cerevisiae* [17, 18].

Apart from being a biotechnologically important organism, *B*. *bruxellensis* can also serve as a model for yeast evolution. It separated from the *S*. *cerevisiae* lineage prior to the lineage-specific whole genome duplication. Interestingly, similar to *S*. *cerevisiae*, it developed a fermentative, Crabtree-positive life-style in a case of parallel evolution, possibly through the loss of a regulatory element affecting expression of genes associated with respiration [19, 20]. Those losses might have been facilitated by partial amplifications of the genome, which relaxed the selective pressure for ordered expression from the amplified genes [21]. Extensive chromosome polymorphisms and–rearrangements in different *B*. *bruxellensis* strains have been demonstrated by pulsed field electrophoresis. Such rearrangements are common in non-sexual species, and therefore, the description of *B*. *bruxellensis* as a sexual species has been called into question [22].

Due to the emergence of next generation sequencing (NGS) methods, a variety of genomes of *B*. *bruxellensis* wine- and beer strains has been sequenced to date [21, 23–29]; however, annotated genomes of isolates from industrial ethanol plants are yet to be reported. The majority of the sequenced genomes seems to be diploid [30]; yet some allotriploid wine strains, containing a third set of chromosomes with a sequence slightly different from the other two chromosomes, have been identified [31]. Chromosome polymorphism has been demonstrated on the level of complete genome sequences in *D*. *bruxellensis* UMY321 wine isolate generated by Nanopore MinION Sequencing [29]. Genome assemblies from short sequencing reads usually produce short scaffolds, making it difficult to follow events of rearrangements, amplifications or deletions of large chromosomal fragments [23–28]. In a recent study, we presented a method that enabled assembly of scaffolds representing chromosomes, using a combination of two complementary sequencing platforms (Illumina, PacBio) and structural mapping provided by the OpGen method [32]. We now annotated the genome of the industrial isolate CBS 11270, enabling genetic analysis to determine ploidy, to understand the distribution of genes over the four identified chromosomes, to identify gene content and possible amplifications and–losses on the chromosomes, and to determine polymorphisms within our strain of interest and when compared to another strain of the same species.

## Materials and methods

### Assembly

The genome assembly was described earlier [32]. However, for the present study, the genome assembly was additionally subjected to manual curation, which is depicted in the Results section.

### Annotation

The annotation of the *B*. *bruxellensis* CBS 11270 genome assembly was performed using the reference annotation of the existing assembly of *B*. *bruxellensis* CBS 2499 and matching annotation, version 2.0, available from the JGI website (http://genome.jgi.doe.gov/vista_embed/?organism=Dekbr2). Gene models were computed using the Maker package (version 2.31.8, PMID: 22192575) based on protein sequences from the reference assembly in combination with a fungi specific repeat library. Rather than simply projecting the existing annotation through syntenic mapping of the scaffolds, this approach re-built the reference annotation on top of our assembly, thus more effectively taking into account any difference in sequence or structure. While we also tried different permutations of RNA-sequences-based annotations, detailed manual inspection indicated that the protein-guided annotation best met our needs with respect to the comparative analyses we wished to perform. Further and more densely sampled transcriptome data may change this view in the future. We used EMBLmyGFF3 tool to deposit genome annotation at European Nucleotide Archive (ENA) [33].

### Repeat analysis

Repeat Masker (http://www.repeatmasker.org) was used with default settings to mask known repeats present in the CBS 11270 genome.

### SNP analysis

Genome sequences’ dictionaries were created using Picard tools version1.107 (http://broadinstitute.github.io/picard/). The Illumina reads [32] were mapped to the reference (genome of *B*. *bruxellensis* CBS 2249) and the new assembly dictionaries by using BWA version 0.7.4 [34]. The files resulting from mapping were, in the case of SAM files, indexed and sorted using samtools version 1.2 [35] and the read coverage was counted for both the reference and the new assembly.

The mapped Illumina reads were run through the GATK HaplotypeCaller version 2.8–1 [36] pipeline, using default settings, to identify the various variants (SNP and indels), and their location and frequency (including allele frequency) present in the reference and the new assembly. We used FreeBayes (1.1.0) for haplotype sampling analysis.

### Gene copy number analysis

The software CNVnator version 0.3 was used to identify copy number variations (CNV) [37].

We set a window size of one hundred for all steps of CNV analysis: generation of histograms of the read depth, calculation of statistical significances for the fragments with unusual read depth, partitioning of the chromosome into regions with similar read depth and CNVs identification.

### Comparative analysis of genome assemblies

Comparative analysis of the genome assemblies of *B*. *bruxellensis* CBS 11270 and CBS 2499 was performed using the Multiple genome alignment tool Mauve version 2.3.1 under default settings of the “Progressive Mauve” function [38].

### Comparative analysis of gene content

Comparison of the gene content between the two genomes was done using program BLASTN 2.2.29+ with a culling limit of one, in order to collect only the best hit, since the objective was to determine presence/absence of homologues [39]. The cut off E value for genes to be considered homologous was 1e-10 [24]. The search for strain specific gene duplications was performed without constraining a culling limit.

BLASTP 2.2.29+ was used for comparison of proteins to identify substitutions of amino acids.

## Results

### Genome structure

**Fig 1:**
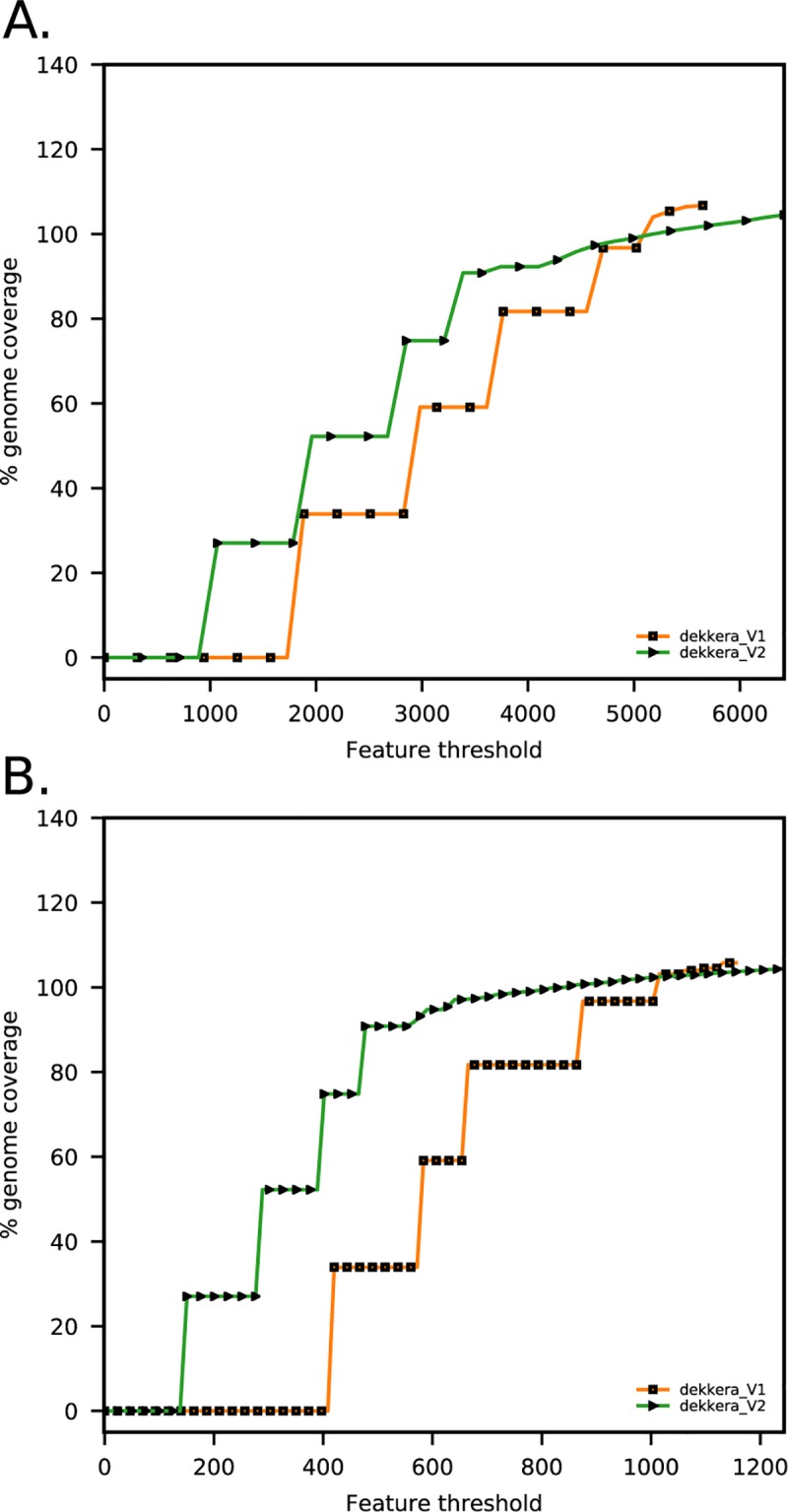
**Feature response curves (FRC) computed for all features (A) and low coverage features (B) (adapted from [32]).** FRC are shown for HGAP, allpaths, dekkera_V1 (final assembly presented in [32]) and dekkera_V2 (assembly presented in this work). The decreased amount of features when removing the duplicated fragment of chromosome 1 in dekkera_V1 assembly is mostly attributed to the loss of regions below normal read coverage (B). Such regions are often indicative of incorrect repeat expansions made by the assembly program [32].

In a previous study [32], the genome assembly of CBS 11270 was demonstrated to be organized in four large chromosomes. Analysis of the *B*. *bruxellensis* genome assembly [32] using BLASTN showed that a 1 Megabase pairs (Mbp) fragment from nucleotide 2,619,547 to 3,634,467 of chromosome 1 was duplicated. Based on coverage information, we verified that this was an assembly artefact and adjusted the assembly by removal of this fragment using a custom R script. This reduced the number of regions in the genome assembly with a lower than average depth of aligned Illumina reads (Fig 1) [40]. The finalized assembly of the CBS 11270 genome consists of four chromosomes, spanning 4 Mbp (chromosome 1), 3.3 Mbp (chromosome 2), 3.7 Mbp (chromosome 3), and 2.2 Mbp (chromosome 4) respectively. The determined chromosome sizes are in line with results from pulsed field electrophoresis [16]. Additionally, 394 contigs of totally 2.1 Mbp (13.7% of the total genome size) were assembled but could not be associated with chromosomes (See Data availability section for accession numbers) [32]. These sequences are, (i) Illumina contigs with no alignments to the optical map assembly (mostly contigs shorter than 40 Kbp), or (ii) unaligned flanks of optical-map-aligned contigs or (iii) flanks or contigs with ambiguous alignments or iv) unique PacBio contigs [41]. The total size of the nuclear genome was thus determined to be 15.3 Mbp, which is comparable to other *B*. *bruxellensis* strains that have been sequenced [21, 23–28]. 97.3% of Illumina reads mapped to the genome assembly draft, of them 88.8% aligned to chromosome sequences and 11.2% to contigs that could not be associated with chromosomes.

A contig of 75 Kbp (scaffold 39309 produced by ABySS), representing mitochondrial DNA was also assembled.

An investigation of heterozygous sites by SNP-analysis showed that the ploidy of CBS 11270 is more than haploid. Average frequency of a particular allele at a heterozygous site in diploid genome is expected to be about 0.5. In a triploid genome partial heterozygous site would have allele frequency of 0.33 or 0.66. The average allele frequency at heterozygous sites was determined to be 0.5 (S1 File), suggesting that the genome of *B*. *bruxellensis* CBS 11270 is diploid. This conclusion is corroborated by results of haplotype sampling analysis (S1 Fig). In contrast to two highly abundant Australian wine strains, AWRI1499 and AWRI1608, additional chromosomes forming an allotriploid hybrid genome [23] were not observed in CBS 11270.

### Genome annotation

**Fig 2:**
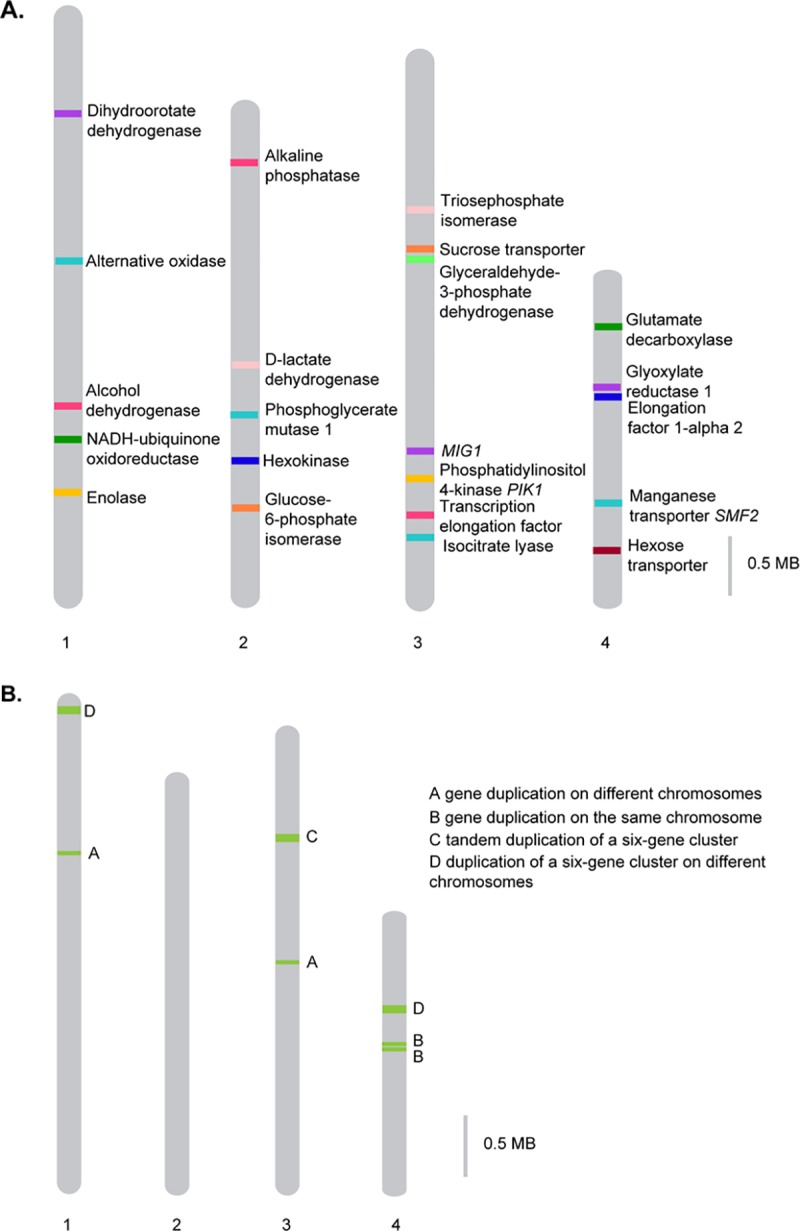
Location of certain genes (A) and duplicated genes (B) on the chromosomes of CBS 11270.

The genome was annotated by using the annotation of *B*. *bruxellensis* CBS 2499 [21] as reference (see Methods). We identified 4897 protein encoding genes (Table 1), which was fewer than in other *B*. *bruxellensis* strains (see below). Chromosome 1 contained 1433 genes; chromosome 2, 1052; chromosome 3, 1191; and chromosome 4, 608 genes. The location of some genes that were discussed in our earlier study [42] is illustrated in Fig 2. Additionally, 595 genes were annotated on contigs not associated with chromosomes, 18 protein encoding genes were detected on the mitochondrial contig.

**Table 1:** Annotation details of the *B*. *bruxellensis* CBS 11270 nuclear genome.

Annotation feature	Counts
Genes	4879
mRNAS	4881
Exons	6342
Introns	1461
Mean introns per mRNA	0.30
Mean intron length	217 bp
Mean CDS length	1358 bp
Mean mRNA length	1423 bp

### Heterozygosity

**Fig 3:**
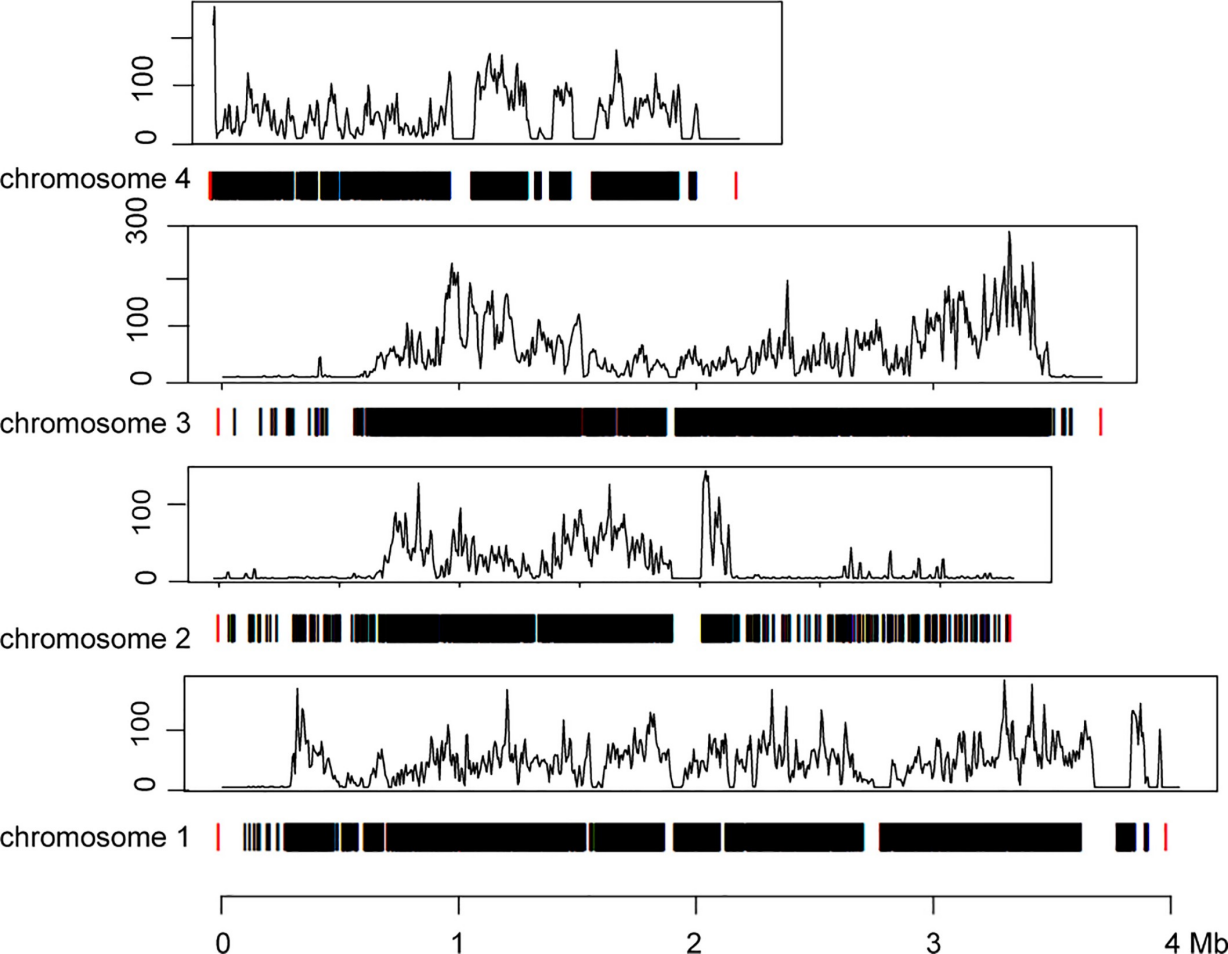
Number and location of variants on chromosomes of *B*. *bruxellensis* CBS 11270. Y-axes represent the number of variants per 10,000 bp. Black bars show occurrence of variants. Red color denotes chromosome margins.

Analysis of polymorphisms between the homologous chromosomes was performed by mapping the CBS 11270 reads to the *de novo* assembly of the CBS 11270 genome. We detected 49,890 single nucleotide polymorphisms (SNPs) (Table 2), constituting 0.32% of the genome size (see S1 and S2 Files). The majority of observed nucleotide variation is due to transitions (i.e. purine-purine or pyrimidine-pyrimidine exchanges), which were observed three times more frequently than transversions (Table 3). Variants were identified in almost all parts of the genome, but with different frequencies at different chromosomal sites (Fig 3). 28,806 variants were detected in non-coding regions (S1 and S2 Files). 21,084 variants occurred in coding sequences, and in total 2668 genes with SNPs were identified (S3 File). 17,423 variants caused amino acid substitutions. The number of variants per gene was highly variable, more than 2,000 genes did not show any SNP (S4 File), 1016 had only 1–3 SNPs. On the other hand, 592 genes had 10 and more SNPs per gene, and 19 of them even had 35 to 75 variants per gene (S1 Table). Some of these genes are shown in Table 4. The normalised by gene length distribution of SNPs per gene Kbp is shown in S2 Fig.

**Table 2:** Statistics of variant analysis in heterozygous sites in genome of CBS 11270 and between genomes of CBS 11270 and CBS 2499.

Variant type	Variant counts in heterozygous sites in genome of CBS 11270	Variant counts between genomes of CBS 11270 and CBS 2499
SNP counts	49890	88534
Indel counts	8538	8133
Total variant counts	58230	96421

**Table 3:** Counts of different types of nucleotide transversions and transitions in heterozygous sites in the genome of CBS 11270 and between the genomes of CBS 11270 and CBS 2499.

SNP type	Counts in the genome of CBS 11270	Counts in the genome of CBS 2499
A/C	3459	5934
A/G	17729	32159
T/G	3547	5845
T/A	4351	7602
C/T	17919	31975
C/G	2890	5036

**Table 4:** Survey of the genes with the highest numbers of SNPs in CBS 11270 and CBS 2499.

Gene ID and position in CBS 11270	Name in CBS 2499	Variants in CBS 11270	Variants in CBS 2499
General negative regulator of transcription subunit 1; BRETBRUG00000001221; chr1: 3243008–3249016	jgi|Dekbr2|64613|fgenesh1_pm.2_#_424	75	102
AP-1 accessory protein *LAA1;* BRETBRUG00000001265; chr1: 3343683–3350348	jgi|Dekbr2|5806|gm1.2215_g	64	108
DNA repair protein *RAD50*; BRETBRUG00000002017; chr2: 1651095–1655015	jgi|Dekbr2|23614|fgenesh1_kg.1_#_362_#_Locus3870v1rpkm14.01	75	84
Protein *SNQ2*; BRETBRUG00000002706; chr3:961178–965875	jgi|Dekbr2|172416|CE84544_34760	63	93
General negative regulator of transcription subunit 2; BRETBRUG00000002734; chr3: 1042035–1042658	jgi|Dekbr2|64613|fgenesh1_pm.2_#_424	11	102

The results of CNV analysis are presented in S5 File and S3 Fig. CNV analysis showed that 372 genes were present on only one of the two homologous chromosomes (S6 File). Most of the genes with reduced copy number were present in clusters (S2 Table). Only 47 of these genes, 17 on chromosome 1, 9 on chromosome 2, 11 on chromosome 3 and 10 on chromosome 4, were not associated with clusters of deleted genes. There was a relatively high number of smaller clusters–two clusters contained two and four genes, and three were formed of seven genes. However, there were also bigger clusters of deleted genes; clusters of 13, 14, 23 and 33 genes with reduced copy number on chromosomes of CBS 11270 were identified. Some clusters were located in close proximity to each other and other clusters were well separated (S2 Table).

**Fig 4:**
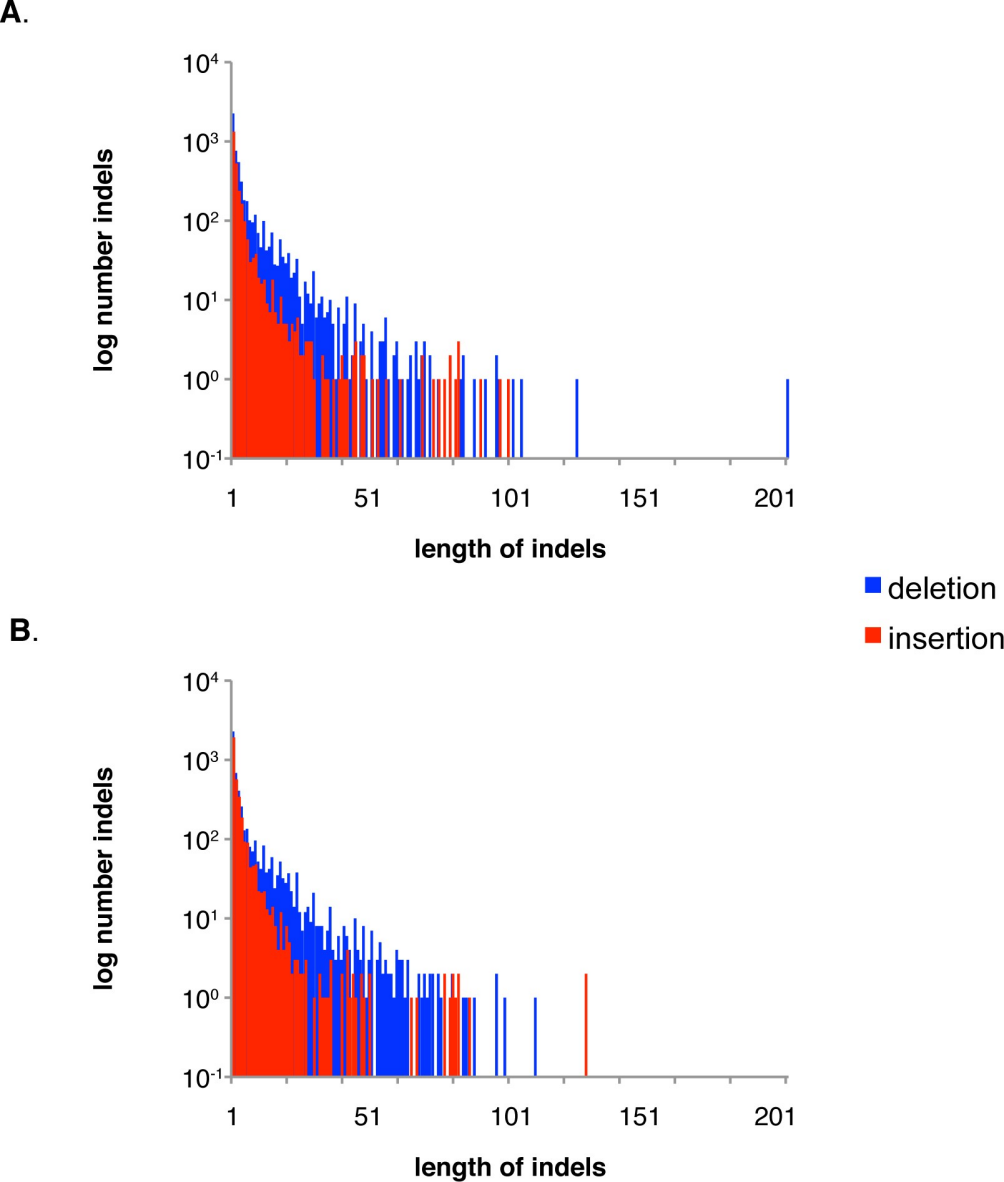
Distribution of indels of different size in heterozygous sites in the genome of CBS 11270 (A) and between genomes of CBS 11270 and CBS 2499 (B).

8538 indels were found in the CBS 11270 genome. We have also found micro/mini satellites in some indels (S1 File). Indels varied in size from 1 to 128 nucleotides. The size of the indels inversely correlated to the frequency: single nucleotide indels occurred 4207 times; indels with a length of 10 nucleotides, 74 times; and indels with 20 nucleotides, 36 times. The longest indel covered 128 nucleotides (Fig 4A). In total, SNPs and indels constituted 58,230 variant counts.

### Gene amplifications

Evidence for amplified genes was investigated by CNV and BLASTN analysis. Twenty genes were found to be duplicated. Six of these genes (Table 5) were found to be duplicated according to copy number analysis only, but were not found in the assembled genome using a BLASTN search. This lack of assembly of duplicated genes is a common problem in genome analysis, typically due to the collapse of repeated regions during assembly [32]. However, most of the amplified genes could be localized to the assembled chromosomes (Table 6, Fig 2). The amplified genes belong to a broad range of GO-categories, including regulation of transcription and replication (e.g. U6 snRNA-associated Sm-like protein LSm8 or histone acetyltransferase *ESA1*), enzymes (e.g. hexokinase-1 or indoleamine C3-dioxygenase), regulators of cellular processes (e.g. flocculation protein gene *FLO5* or temperature shock-inducible protein 1), and transport (e.g. allantoin permease or GABA-specific permease, but no sugar transporters).

**Table 5:** Duplicated genes revealed by CNV analysis and not identified in the genome assembly.

Gene name	Gene ID and location
F0249 protein VIBHAR	BRETBRUG00000000385
	chr1 1018607 1018993
Probable NADPH dehydrogenase	BRETBRUG00000002123
	chr2 2039150 2040358
Chromatin modification-related protein *EAF1*	BRETBRUG00000002201
	chr2 2253321 2256215
Copia protein	BRETBRUG00000002416
	chr2 2815514 2816992
Copia protein	BRETBRUG00000002417
	chr2 2817465 2818151
Repressor of filamentous growth	BRETBRUG00000003666
	chr3 3312856 3314625

**Table 6:** List of duplicated genes resolved by genome assembly.

	Gene name	Gene ID and location		
A	tRNA (guanine(10)-N2)-methyltransferase	BRETBRUG00000001038	BRETBRUG00000003060	
		chr1 2703203 2705172	chr3 1805200 1807169	
B	Methylthioribulose-1-phosphate dehydratase	BRETBRUG00000003998	BRETBRUG00000004000	
		chr4 1127888 1128565	chr4 1129334 1130011	
C	Hexokinase-1	BRETBRUG00000001423	BRETBRUG00000004091	
		chr1 3856343 3857854	chr4 1381931 1383442	
	Flocculation protein *FLO5*	BRETBRUG00000001424	BRETBRUG00000004090	
		chr1 3864230 3868254	chr4 1371498 1375532	
	Temperature shock-inducible protein 1	BRETBRUG00000001425	BRETBRUG00000004089	
		chr1 3870223 3870528	chr4 1369224 1369529	
	Putative transcriptional regulatory protein	BRETBRUG00000001426	BRETBRUG00000004088	
		chr1 3874728 3876680	chr4 1363072 1365024	
	Uncharacterized transcriptional regulatory protein	BRETBRUG00000001427	BRETBRUG00000004087	
		chr1 3877690 3879264	chr4 1360488 1362062	
	Allantoin permease	BRETBRUG00000001428	BRETBRUG00000004086	
		chr1 3880591 3882306	chr4 1357446 1359161	
D	Indoleamine 2 2C3-dioxygenase	BRETBRUG00000003493	BRETBRUG00000003500	BRETBRUG00000003492
		chr3 2804460 2805389	chr3 2823355 2824602	chr3 2801423 2802706
	Histone acetyltransferase *ESA1*	BRETBRUG00000003494	BRETBRUG00000003501	
		chr3 2807345 2808712	chr3 2826194 2827561	
	Serine/threonine-protein phosphatase 2A activator	BRETBRUG00000003495	BRETBRUG00000003502	
		chr3 2808829 2809905	chr3 2827678 2828226	
	GABA-specific permease	BRETBRUG00000003496	BRETBRUG00000003503	
		chr3 2810290 2811957	chr3 2829138 2830805	
	V-type proton ATPase subunit a 2C vacuolar isoform	BRETBRUG00000003497	BRETBRUG00000003504	
		chr3 2813515 2816040	chr3 2832363 2834888	
	Protein *PNS1*	BRETBRUG00000003498	BRETBRUG00000003505	
		chr3 2816967 2818574	chr3 2835815 2837422	

Letter in first column indicates guide-mark in Fig 2B.

Amplification of genes on either the same or on different chromosomes was observed. Interchromosomal single gene duplication was observed for a gene encoding for tRNA (guanine(10)-N2)-methyltransferase (letter A in Table 6 and Fig 2). This gene has copies on chromosome 1 and chromosome 3. One intrachromosomal single gene duplication was identified on chromosome 4 (a gene coding for methylthioribulose-1-phosphate dehydratase, letter B in Table 6 and Fig 2). The methylthioribulose-1-phosphate dehydratase gene copies are separated by the gene encoding for U6 snRNA-associated Sm-like protein LSm8.

There were also two examples of amplified gene clusters. These clusters both contain six genes. One of the clusters (letter C in Table 6 and Fig 2) contains genes encoding for hexokinase-1, flocculation protein *FLO5*, temperature shock-inducible protein 1, putative transcriptional regulatory protein, an uncharacterized transcriptional regulatory protein and allantoin permease. One copy of this cluster is located in chr1:3,856,343–3,882,306 and the other copy in chr4:1,383,442–1,357,446. The other six-gene cluster (letter D in Table 6 and Fig 2) comprises genes encoding for indoleamine C3-dioxygenase, histone acetyltransferase *ESA1*, serine/threonine-protein phosphatase 2A activator, GABA-specific permease, V-type proton ATPase subunit (vacuolar isoform) and protein PNS1. This gene cluster forms a tandem copy chr3:2,804,460–2,818,574 and chr3:2,823,355–2,837,422. Interestingly, in the first copy of the gene cluster, the gene encoding for indoleamine C3-dioxygenase forms itself a tandem duplication (one more copy of this gene is present between chr3:2,801,423–2,802,706), but the gene is not duplicated in the other copy of the gene cluster.

It is notable that none of the duplicated genes in the genome of strain CBS 11270 had analogous duplications in the genome of the wine strain CBS 2499 [21].

### Centromers, simple, low complexity and interspersed repeat analysis

Analysis of centromere structures on chromosomes of *B*. *bruxellensis* is presented in S3 Table. Partial sequences of *B*. *bruxellensis* CBS 2499 centromeres [43] were identified on chromosome 1 (*CEN1*), and on chromosomes 1, 2, and 4 (*CEN2*). None of the identified centromer structures were found on chromosome 3.

Repeats identified in the CBS 11270 genome are summarized in S7 File. 2% of the genome consisted of repeats. We found 53 non-long terminal repeat- (LTR) retrotransposons (48 Long Interspersed Nuclear Elements (LINE) and five Short Interspersed Nuclear Elements (SINE)), 6 DNA transposons (DNA/TcMar-Tigger, DNA/hAT-Ac, DNA/hAT-Charlie), 3228 simple repeats, 771 low complexity repeats, two snRNA, five rRNA and 55 tRNA.

Interestingly, five of the duplicated genes (Table 7) did contain mainly simple and low complexity repeats both in the upstream and downstream flanking regions. Two of these genes (both coding for GABA-specific permease) even contained the non-LTR retrotransposon AmnL2-1 LINE/L2 in the downstream regions. Three of the duplicated genes contained repeats only in downstream regions and two others had retrotransposon AmnL2-1 LINE/L2 in their upstream regions.

**Table 7:** Characterization of repeats in flanking regions of duplicated genes.

Gene id and location	Gene name	Downstream repeat	Upstream repeat
BRETBRUG00000003060	tRNA (guanine(10)-N2)-methyltransferase	(AAGATAG)n Simple_repeat 1807186 1807233	(T)n Simple_repeat 1805044 1805068
chr3 1805200 1807169		(CTC)n Simple_repeat 1807517 1807558	
		(AGTAA)n Simple_repeat 1808962 1809011	
BRETBRUG00000003500	Indoleamine 2 2C3-dioxygenase	A-rich Low_complexity 2826505 2826555	
chr3 2823355 2824602			
BRETBRUG00000003494	Histone acetyltransferase *ESA1*	A-rich Low_complexity 2807656 2807706	
chr3 2807345 2808712			
BRETBRUG00000003495	Serine/threonine-protein phosphatase 2A activator	(T)n Simple_repeat 2810049 2810080	A-rich Low_complexity 2807656 2807706
chr3 2808829 2809905			
BRETBRUG00000003496	GABA-specific permease	AmnL2-1 LINE/L2 2812187 2812238	(T)n Simple_repeat 2810049 2810080
chr3 2810290 2811957			
BRETBRUG00000003497	V-type proton ATPase subunit a 2C vacuolar isoform		AmnL2-1 LINE/L2 2812187 2812238
chr3 2813515 2816040			
BRETBRUG00000003501	Histone acetyltransferase *ESA1*	(T)n Simple_repeat 2828899 2828928	
chr3 2826194 2827561			
BRETBRUG00000003502	Serine/threonine-protein phosphatase 2A activator	(T)n Simple_repeat 2828899 2828928	A-rich Low_complexity 2826505 2826555
chr3 2827678 2828226			
BRETBRUG00000003503	GABA-specific permease	AmnL2-1 LINE/L2 2831034 2831085	(T)n Simple_repeat 2828899 2828928
chr3 2829138 2830805			
BRETBRUG00000003504	V-type proton ATPase subunit a%2C vacuolar isoform		AmnL2-1 LINE/L2 2831034 2831085
chr3 2832363 2834888			

### Comparative genome analysis of *B*. *bruxellensis* CBS 11270 and CBS 2499

To compare the genome organization of CBS 11270 with another *B*. *bruxellensis* strain, we aligned the scaffolds obtained for the wine strain CBS 2499 [21] to the chromosomes of CBS 11270 (S4 Fig) using the multiple genome alignment tool Mauve 2.3.1 (see Methods). This program recognizes regions similar to the reference as blocks. Those regions could be composed of several scaffolds when they align to a larger reference scaffold, or they can also be part of a scaffold, if the rest of the scaffold does not align at this position. Scaffolds 4, 6, 27 and a substantial part of scaffold 2 form a block with high similarity to the segment between 0.1 Mbp to 2.5 Mbp of chromosome 1. Moreover, also a part of scaffold 3 mapped to chromosome 1. Two blocks of scaffold 2 had another order as compared to the homologous regions of chromosome 1. Scaffolds 17, 1, 29, 15 and 12 almost completely covered chromosome 2. The first segment of chromosome 3 up to 1.6 Mbp was almost completely covered by scaffolds 18, 5, 8 and 14. Apart from this, parts of scaffolds 2, 3 and 13 mapped to chromosome 3. Major parts of scaffolds 20, 10, 16, 21, 7, 11, 9 and 24 were similar to parts of chromosome 4, whereas only the last third of scaffold 19 aligned to chromosome 4.

We also investigated single nucleotide polymorphisms (SNPs) and indels between the two strains, by mapping the CBS 11270 reads to the CBS 2499 genome (see Methods and S8 File). CBS 11270 differed from CBS 2499 in 96.421 variants: 88.534 SNPs and 8.133 indels. 10.626 variants are homozygous, 85.552 heterozygous with one allele common to CBS 2499 and 243 are potential heterozygous variants. with both alleles different from CBS2499.

42,064 inter-strain variants were located inside open reading frames (ORF), i.e. 47.5% of total inter-strain variants are in coding regions, which is higher than the proportion of heterozygous sites in ORFs of CBS 11270 (38%). 28,679 variants caused amino acid substitution. Almost all genes (4,410) were polymorphic between *B*. *bruxellensis* CBS 11270 and CBS 2499. 46,450 variants were in non-coding regions (see S9 File). A list of the genes containing variants and a list of genes without variants is presented in S10 and S11 Files, respectively. As observed in the intra-strain heterozygosity pattern (see above), transitions were three times more abundant than transversions, and the number of variants per gene and gene counts were in inverse relationship. 839 genes were found with one variant. In one gene, annotated as gm1.2215_g (AP-1 accessory protein) (Table 4), 108 variants were found. Other genes with a high number of variants included: fgenesh1_pm.2_#_424 b (Ccr4-Not transcription complex subunit (NOT1) 100 variants, CE91624_56964 (hypothetical protein) 97 variants, CE84544_34760 (multidrug transporter) 87 variants, fgenesh1_kg.1_#_362_#_Locus3870v1rpkm14.01 (DNA repair protein RAD50) 84 variants, e_gw1.2.1034.1 (Midasin) 76 variants, estExt_Genewise1Plus.C_5_t20257 (RNA helicase) 75 variants, gm1.2263_g (hypothetical protein) 69 variants, estExt_Genewise1Plus.C_6_t20136 (N-glycosylated protein) 68 variants, gm1.360_g (protein of unknown function) 63 variants.

The size of the indels ranged from 1 to 201 nucleotides (Fig 4B). Indels of almost all sizes were most often sequences from CBS 2499 absent in CBS 11270 rather than the opposite. 3,571 single nucleotide indels were found in CBS 11270 compared to CBS 2499. In total, 8,133 indels were observed in CBS 11270 compared to CBS 2499. These 8,133 indels had a total length of 41,233 nucleotides.

### Gene content differences between CBS 11270 and CBS 2499

CBS 11270 and CBS 2499 differed in their gene contents. 19 genes were found in CBS 2499 but not in CBS 11270, by using BLASTN-search versus whole CBS 11270 genome assembly (see S4 Table). Table 8 shows some of these genes present in the genome of CBS 2499 but absent in CBS 11270. Most of these genes are hypothetical proteins. Two genes involved in transport through plasma membrane, a gene encoding for Na+/H+ antiporter involved in sodium and potassium efflux and a putative transmembrane sensor transporter were absent from the CBS 11270 genome. A gene involved in antioxidant metabolism, s-formylglutathione hydrolase was also absent in CBS 11270. A gene coding for maltase was absent in CBS 11270, which is not consistent with the ability of this strain to grow on maltose [16].

**Table 8:** Genes in the *B*. *bruxellensis* CBS 2499 genome absent in the CBS 11270 genome.

Gene name	Best blast hit
jgi|Dekbr2|26744|fgenesh1_kg.19_#_10_#_Locus2611v2rpkm45.30	Na+/H+ antiporter involved in sodium and potassium efflux through the plasma membrane [*Ogataea parapolymorpha* DL-1].
jgi|Dekbr2|8850|gm1.5259_g	maltase [*Brettanomyces bruxellensis* AWRI1499].
jgi|Dekbr2|8855|gm1.5264_g	putative transmembrane sensor transporter [*Brettanomyces bruxellensis* AWRI1499].
jgi|Dekbr2|145681|CE57809_24 NA jgi|Dekbr2|51831|e_gw1.23.15.1	s-formylglutathione hydrolase [*Brettanomyces bruxellensis* AWRI1499]

31 genes were identified in CBS 11270 that were not present in CBS 2499 (S5 Table). Further analysis would be required to verify the absence of these genes in CBS 2499.

## Discussion

This study represents the first genomic investigation of a *B*. *bruxellensis*-strain that functions as an ethanol production strain [12, 16]. Using the recently developed assembly of the CBS 11270 genome to scaffolds of chromosome size [44] we could associate a major part, 86.4% of the genome sequences, to the assembled four chromosomes.

Due to the re-construction of chromosomes we could identify larger re-arrangements of the genome, and we found that the *B*. *bruxellensis*-genome is highly flexible. Scaffolds identified earlier in the wine isolate CBS 2499 [21] were split or arranged differently in CBS 11270. For instance, parts of scaffold 2 of CBS 2499 mapped to chromosomes 1 and 3 in CBS 11270, and parts of scaffold 2 were in a different order compared to CBS 2499. Alternatively, the mismatch of contigs order between two genomes could arise from assembly errors [45]. The combination of various sequencing and assembly strategies aimed to strengthen the accuracy of the CBS 11270 genome sequence [32]. The size of our identified four chromosomes was in the range from 2.2–4 Mbp, which fits to results obtained by pulsed field electrophoresis. The pulsed field investigations even indicated a potential fifth chromosome of about 500 kb [18], and it is possible that some of our non-assembled contigs belong to this chromosome. However, using our assembly approach we could not confirm its existence [32]. Large differences between different *B*. *bruxellensis* strains in chromosome size and -number have been demonstrated by pulsed field electrophoresis, with chromosome sizes ranging from below 1 Mbp up to 6 Mbp, and chromosome numbers up to nine [22]. We also found a number of deletions (in the largest case about 149099 bp were missing in one of the homologues of chromosome 1, leading to a deletion of 33 genes) in homologous chromosomes. These findings strongly indicate a very flexible genome of *B*. *bruxellensis*. Chromosome re-arrangements have mainly been observed in non-sexual species such as *Candida glabrata* or *Candida albicans* [46, 47]. Ordered meiosis seems to be difficult or impossible when there is such flexibility of chromosomes. Ascospores have been observed in *B*. *bruxellensis* [48], but no further investigation of those ascospores has been reported, and thus there is no genetic evidence for the existence of a sexual cycle in *B*. *bruxellensis*. On the other hand, the existence of allotriploid wine strains indicates mating activity even over species borders [23, 26]. Possibly, *B*. *bruxellensis* uses a similar program of genetic recombination as has been described for *C*. *albicans*, where mating is followed by a mitotic chromosome loss [49].

In general, we found a very high variability in the genome of the industrial strain. The number of SNPs when comparing the homologous chromosomes (44,022, i.e. 0.34% of the total haploid genome) was higher than the variability between distantly related *S*. *cerevisiae* strains. In *S*. *cerevisiae* the number of variants is lower and varies between strains: 39, 4894, 7955, 13,914, 25,298 between S288C and BY4716, A364A, W303, FL100, CEN.PK, S1278b, SK1 [50], YJSH1 [51], respectively. Curtins et al. reported 342,900 heterozygotic sites within the genome of the wine isolate AWRI1499 [26]. Distribution of SNPs along the chromosomes was uneven, with local maxima of the SNP-frequence (Fig 3), indicating the location of highly polymorphic sequences or highly repetitive sequences, similar to that observed for chromosomes of *S*. *cerevisiae* [50–52].

There was a considerable interstrain- variability, more than 88,000 SNPs were identified in CBS 11270 compared to the wine strain CBS 2499. In total, 96,421 variants (SNPs and indels) were found between the two strains. This was slightly higher but still in the same order of what has been found when comparing several wine strains, ST05.12/22 and AWRI 1499 (79,627 variants), and ST05.12/22 and CBS 2499 (82,676 variants) These numbers illustrate, that there is a high diversity within the species *B*. *bruxellensis*.

Among the SNPs, transitions were about three times as frequent as transversions. Although there are double as many possibilities for transversions to occur, the transition to transversion bias has been observed in almost all known biological systems. Transitions are only in half the cases resulting in amino acid exchanges compared to transversions, however, as it has recently been pointed out, the background of the transition:transversion bias is not really understood [53].

We found 18 genes with high SNP density (more than 35 SNPs per gene), suggesting that they may be under some selective pressure (Weihong Qi 2009) [54]. Indeed, Yi-Cheng Guo et al (2016) showed that the genes of the transcription system in *B*. *bruxellensis* CBS 2249 exhibited faster evolution than other genes [55]. We identified 77 SNPS in a gene coding for a general negative regulator of transcription subunit 1 (BRETBRUG00000002734) in CBS 11270 (see Table 4).

The ecosystem from which this strain has been isolated is very different from that of wine and beer strains [13, 44, 56]. The industrial conditions, consisting of year-long continuous cultivation with cell recirculation at constant low pH (3.5), considerable ethanol concentrations (about 60 g/l), and relatively high temperature (37°C) [12], provide a stressful, but relatively constant environment. Constant environments often result in reductive evolution, resulting in gene losses within the strains under these conditions [29, 56]. Frequently, loss-of-function mutations can provide a selection advantage in those environments [57]. However, although we observed a substantial loss of heterozygosity, i.e. loss of one of the homologous genes in 372 cases, we did not find a substantial loss of function within known metabolic pathways. Massive loss of heterozygosity was also shown in *D*. *bruxellensis* wine strain UMY321 [29]. There may be various challenges for the strain in the ethanol process, for instance during cell recirculation, or when interacting with the high number of lactic acid bacteria in the process [10, 58], which provide a certain selectivity for multiple metabolic pathways. Previous experiments showed that isolates from this process are able to ferment cellobiose [14], and that CBS 11270 can adapt to inhibitors of lignocellulose hydrolysate [18] and thus can cope with conditions that are quite different from a starch-based ethanol process. In diploids, events other than merely gene losses, such as mutations modifying gene expression, may provide a fitness advantage for the respective strain [49], and further investigation may be required to identify mutations that are specific for the ethanol production environment.

*B*. *bruxellensis* is a unique yeast with an amazing competitiveness in the stressful environments of wine-, beer- and bioethanol production. Many traits of its physiology are still not understood. A variety of isolates from wine and beer production have been sequenced to date. Here, we present the first genome of an ethanol production strain in chromosome-sized scaffolds which may serve as a reference to reconstruct chromosomes of strains from a variety of environments. This will help to reconstruct mutational events that are correlated to the adaptation to different environments, and thus, contribute to understanding of the unique features of *B*. *bruxellensis* physiology. Moreover, our study demonstrates the enormous flexibility of the *B*. *bruxellensis* genome. This flexibility may be utilized in artificial evolution experiments in appropriate long-term cultivations, and thus, together with the recently developed methods for genetic manipulation of this yeast [49], provide a tool for obtaining strains for future biotechnological applications [13, 44, 56].

## Supporting information

S1 File Variants in CBS 11270. (TXT)

S2 File Characterization of variants in CBS 11270. (TXT)

S3 File List of genes without variants in CBS 11270. (TXT)

S4 File List of genes in CBS 11270 with variants. (TXT)

S5 File Copy number variation in CBS 11270. (TXT)

S6 File List of genes with reduced coverage. (TXT)

S7 File Characterization of repeats in CBS 11270. (TXT)

S8 File Variants in CBS 2499. (TXT)

S9 File Characterization of variants in CBS 2499. (TXT)

S10 File List of genes in CBS 2499 with variants. (TXT)

S11 File List of genes without variants in CBS 2499. (TXT)

S1 Table Distribution of genes with different numbers of variants in heterozygous sites in the genome of CBS 11270 and between genomes of CBS 11270 and CBS 2499. (DOCX)

S2 Table Clustering of genes with reduced coverage in CBS 11270. (DOCX)

S3 Table Position of centromeres. (DOCX)

S4 Table List of genes present in CBS 2499 but not in CBS 11270. (DOCX)

S5 Table List of genes present in CBS 11270 but not in CBS 2499. (DOCX)

S1 Fig Haplotype sampling of CBS 11270. (DOCX)

S2 Fig Distribution of SNPs per Kbp gene. (DOCX)

S3 Fig Variation in coverage of chromosomes in CBS 11270. (DOCX)

S4 Fig Mauve alignment. (DOCX)
