# Supplementary material for: Chromosomal genome assembly of the ethanol production strain CBS 11270 indicates a highly dynamic genome structure in the yeast species Brettanomyces bruxellensis
Source: PLoS One. 2019 May 1;14(5):e0215077. doi: 10.1371/journal.pone.0215077 (PMC6493715; doi:10.1371/journal.pone.0215077)
Supplement: S1 Table — (DOCX) [file pone.0215077.s012.docx]

**S1 Table. Distribution of genes with different numbers of variants in heterozygous sites in the genome of CBS 11270 and between genomes of CBS 11270 and CBS 2499.**

| Number of variants per gene | CBS 11270 | CBS 2499 |
| --- | --- | --- |
| 108 |  | 1 |
| 102 |  | 1 |
| 100 |  | 1 |
| 97 |  | 1 |
| 96 |  | 1 |
| 95 |  |  |
| 93 |  | 1 |
| 88 |  | 1 |
| 87 |  | 2 |
| 86 |  | 1 |
| 84 |  | 1 |
| 81 |  |  |
| 78 |  |  |
| 77 |  | 2 |
| 76 |  |  |
| 75 | 2 |  |
| 74 |  | 2 |
| 73 |  |  |
| 70 |  |  |
| 69 |  | 1 |
| 68 |  |  |
| 67 |  | 1 |
| 65 |  | 3 |
| 64 | 1 | 1 |
| 63 | 1 | 2 |
| 62 |  | 2 |
| 61 | 1 | 5 |
| 60 |  | 1 |
| 59 |  | 4 |
| 58 | 1 |  |
| 57 |  | 4 |
| 56 |  | 3 |
| 55 |  | 3 |
| 54 | 2 | 1 |
| 53 | 2 | 4 |
| 52 | 1 | 4 |
| 51 |  |  |
| 50 |  | 6 |
| 49 | 3 | 5 |
| 48 | 1 | 4 |
| 47 | 1 | 9 |
| 46 | 1 | 10 |
| 45 |  | 5 |
| 44 |  | 7 |
| 43 |  | 7 |
| 42 |  | 5 |
| 41 | 2 | 6 |
| 40 | 1 | 8 |
| 39 | 1 | 11 |
| 38 |  | 17 |
| 37 | 1 | 10 |
| 36 |  | 15 |
| 35 | 3 | 23 |
| 34 | 5 | 16 |
| 33 | 4 | 16 |
| 32 | 3 | 38 |
| 31 | 2 | 21 |
| 30 | 5 | 26 |
| 29 | 2 | 20 |
| 28 | 8 | 30 |
| 27 | 6 | 27 |
| 26 | 8 | 33 |
| 25 | 12 | 39 |
| 24 | 15 | 45 |
| 23 | 11 | 44 |
| 22 | 10 | 40 |
| 21 | 17 | 50 |
| 20 | 21 | 65 |
| 19 | 15 | 53 |
| 18 | 26 | 77 |
| 17 | 20 | 79 |
| 16 | 36 | 77 |
| 15 | 36 | 107 |
| 14 | 28 | 121 |
| 13 | 56 | 115 |
| 12 | 58 | 140 |
| 11 | 81 | 142 |
| 10 | 85 | 173 |
| 9 | 87 | 168 |
| 8 | 125 | 203 |
| 7 | 165 | 217 |
| 6 | 164 | 226 |
| 5 | 254 | 254 |
| 4 | 279 | 324 |
| 3 | 324 | 343 |
| 2 | 510 | 394 |
| 1 | 483 | 485 |
