# Supplementary material for: Chromosomal genome assembly of the ethanol production strain CBS 11270 indicates a highly dynamic genome structure in the yeast species Brettanomyces bruxellensis
Source: PLoS One. 2019 May 1;14(5):e0215077. doi: 10.1371/journal.pone.0215077 (PMC6493715; doi:10.1371/journal.pone.0215077)
Supplement: S2 Table — (DOCX) [file pone.0215077.s013.docx]

**S5 Table. Clustering of genes with reduced coverage in CBS 11270.**

| Chromosome | Number of single gene deletions | Clustered gene deletions |
| --- | --- | --- |
| chr1 | 17 | 2 gene cluster: 1819401 1822900  33 gene cluster 2632901-2782000  8-gene cluster 3855001-3883600  4-gene cluster 3885501-3897500 |
| chr2 | 9 |  |
| chr3 | 11 | 2-gene cluster: 1455401-1461000  13-gene cluster: 1794401-1832300  14-gene cluster: 1832601-1864100  7-gene cluster: 1864301-1920100  8-gene cluster: 2803101-2823500  7-gene cluster: 2825001-2842100 |
| chr4 | 10 | 7-gene cluster: 618701-625100  4-gene cluster: 1127801-1130600  23-gene cluster: 1330501-1429300 |
