# Supplementary material for: Chromosomal genome assembly of the ethanol production strain CBS 11270 indicates a highly dynamic genome structure in the yeast species Brettanomyces bruxellensis
Source: PLoS One. 2019 May 1;14(5):e0215077. doi: 10.1371/journal.pone.0215077 (PMC6493715; doi:10.1371/journal.pone.0215077)
Supplement: S3 Table — (DOCX) [file pone.0215077.s014.docx]

**S2 Table. Position of centromeres.**

| Centromere type | Length of overlap (bp) | Start of centromere sequence overlap | Stop of centromere sequence overlap | Start of chromosome overlap | Stop of chromosome overlap |
| --- | --- | --- | --- | --- | --- |
| CEN2 | 1919 | 6 | 1921 | chr1:1165671 | chr1:1163756 |
| CEN2 | 483 | 2120 | 2602 | chr1:1163758 | chr1:1163276 |
| CEN2 | 196 | 1925 | 2120 | chr2:2251006 | chr2:2251201 |
| CEN2 | 196 | 1925 | 2120 | chr4:972731 | chr4:972536 |
| CEN1 | 822 | 23 | 844 | chr1:1022893 | chr1:1022072 |
