# Supplementary material for: Chromosomal genome assembly of the ethanol production strain CBS 11270 indicates a highly dynamic genome structure in the yeast species Brettanomyces bruxellensis
Source: PLoS One. 2019 May 1;14(5):e0215077. doi: 10.1371/journal.pone.0215077 (PMC6493715; doi:10.1371/journal.pone.0215077)
Supplement: S4 Table — (DOCX) [file pone.0215077.s015.docx]

**S4 Table. List of genes present in CBS 2499 but not in CBS 11270.**

| Gene name | Gene id |
| --- | --- |
| jgi\|Dekbr2\|24144\|fgenesh1_kg.2_#_63_#_Locus2970v1rpkm39.61 | hypothetical protein TATV_DAH68_216 [Taterapox virus] |
| jgi\|Dekbr2\|24425\|fgenesh1_kg.2_#_344_#_Locus3171v6rpkm0.13_PRE | NA |
| jgi\|Dekbr2\|146111\|CE58239_13409 | hypothetical protein JL09_g3854 [Pichia kudriavzevii] |
| jgi\|Dekbr2\|5574\|gm1.1983_g | hypothetical protein JL09_g3854 [Pichia kudriavzevii] |
| jgi\|Dekbr2\|7199\|gm1.3608_g | NA |
| jgi\|Dekbr2\|7232\|gm1.3641_g | hypothetical protein PBRA_008756 [Plasmodiophora brassicae] |
| jgi\|Dekbr2\|26745\|fgenesh1_kg.19_#_11_#_Locus5793v2rpkm0.11 | NA |
| jgi\|Dekbr2\|51944\|e_gw1.65.2.1 | hypothetical protein NEMVEDRAFT_v1g225628 [Nematostella vectensis] |
| jgi\|Dekbr2\|51948\|e_gw1.65.1.1 | hypothetical protein AGABI2DRAFT_75878 [Agaricus bisporus var. bisporus H97] |
| jgi\|Dekbr2\|8922\|gm1.5331_g | unnamed protein product [Cyberlindnera jadinii] |
| jgi\|Dekbr2\|67592\|fgenesh1_pm.75_#_1 | hypothetical protein CaO19.6835 [Candida albicans SC5314] |
| jgi\|Dekbr2\|51958\|e_gw1.75.3.1 | hypothetical protein PICST_57317, partial [Scheffersomyces stipitis CBS 6054] |
| jgi\|Dekbr2\|181898\|CE94026_164653 | hypothetical protein QG37_01684 [Candida auris] |
| jgi\|Dekbr2\|156990\|CE69118_488 | PREDICTED: S-antigen protein-like, partial [Salmo salar] |
| jgi\|Dekbr2\|26744\|fgenesh1_kg.19_#_10_#_Locus2611v2rpkm45.30 | Na+/H+ antiporter involved in sodium and potassium efflux through the plasma membrane [Ogataea parapolymorpha DL-1] |
| jgi\|Dekbr2\|8850\|gm1.5259_g | maltase [Brettanomyces bruxellensis AWRI1499] |
| jgi\|Dekbr2\|8855\|gm1.5264_g | putative transmembrane sensor transporter [Brettanomyces bruxellensis AWRI1499] |
| jgi\|Dekbr2\|145681\|CE57809_24 | NA |
| jgi\|Dekbr2\|51831\|e_gw1.23.15.1 | s-formylglutathione hydrolase [Brettanomyces bruxellensis AWRI1499] |
