# Supplementary material for: Chromosomal genome assembly of the ethanol production strain CBS 11270 indicates a highly dynamic genome structure in the yeast species Brettanomyces bruxellensis
Source: PLoS One. 2019 May 1;14(5):e0215077. doi: 10.1371/journal.pone.0215077 (PMC6493715; doi:10.1371/journal.pone.0215077)
Supplement: S5 Table — (DOCX) [file pone.0215077.s016.docx]

**S5 Table. List of genes present in CBS 11270 but not in CBS 2499.**

| Gene name | Gene id |
| --- | --- |
| acyl-coenzyme A oxidase | DEKBRUBILSv1T102354 |
| melanopsin | DEKBRUBILSv1T101096 |
| Bromoperoxidase-catalase | dekbrubilsv2t00000000659 |
| Serine/threonine-protein kinase KIC1 | dekbrubilsv2t00000000506 |
| Succinyl-CoA ligase [ADP-forming] subunit alpha | dekbrubilsv2t00000000570 |
| 60 kDa chaperonin 2 | dekbrubilsv2t00000000115 |
| Elongation factor 4 | dekbrubilsv2t00000000626 |
| NADH-quinone oxidoreductase subunit B | dekbrubilsv2t00000000746 |
| Superoxide dismutase [Mn] | dekbrubilsv2t00000000566 |
| Probable cytochrome c oxidase subunit 1-alpha | dekbrubilsv2t00000000700 |
| hypothetical protein | dekbrubilsv2t00000000449 |
| Isocitrate dehydrogenase [NADP] | dekbrubilsv2t00000000365 |
| Elongation factor G | dekbrubilsv2t00000000398 |
| Elongation factor Tu | dekbrubilsv2t00000000662 |
| Succinate dehydrogenase flavoprotein subunit | dekbrubilsv2t00000000722 |
| Succinate dehydrogenase iron-sulfur subunit | dekbrubilsv2t00000000145 |
| Chaperone protein DnaK | dekbrubilsv2t00000000603 |
| Glyceraldehyde-3-phosphate dehydrogenase | dekbrubilsv2t00000000696 |
| Glucose-6-phosphate isomerase | dekbrubilsv2t00000000542 |
| Thioredoxin reductase | dekbrubilsv2t00000000188 |
| NADH-quinone oxidoreductase subunit H | dekbrubilsv2t00000000337 |
| ATP synthase subunit beta | dekbrubilsv2t00000000697 |
| ATP synthase subunit alpha | dekbrubilsv2t00000000087 |
| hypothetical protein | dekbrubilsv2t00000000433 |
| Enolase | dekbrubilsv2t00000000285 |
| Guanine nucleotide-binding protein subunit alpha | dekbrubilsv2t00000000156 |
| Adenylosuccinate synthetase | dekbrubilsv2t00000000032 |
| Pyridoxal 5'-phosphate synthase subunit PdxS | dekbrubilsv2t00000000089 |
| 50S ribosomal protein L14 | dekbrubilsv2t00000000140 |
| 30S ribosomal protein S19 | dekbrubilsv2t00000000003 |
| 30S ribosomal protein S12 | dekbrubilsv2t00000000131 |
