# Supplementary material for: Chromosomal genome assembly of the ethanol production strain CBS 11270 indicates a highly dynamic genome structure in the yeast species Brettanomyces bruxellensis
Source: PLoS One. 2019 May 1;14(5):e0215077. doi: 10.1371/journal.pone.0215077 (PMC6493715; doi:10.1371/journal.pone.0215077)
Supplement: S1 Fig — (DOCX) [file pone.0215077.s017.docx]

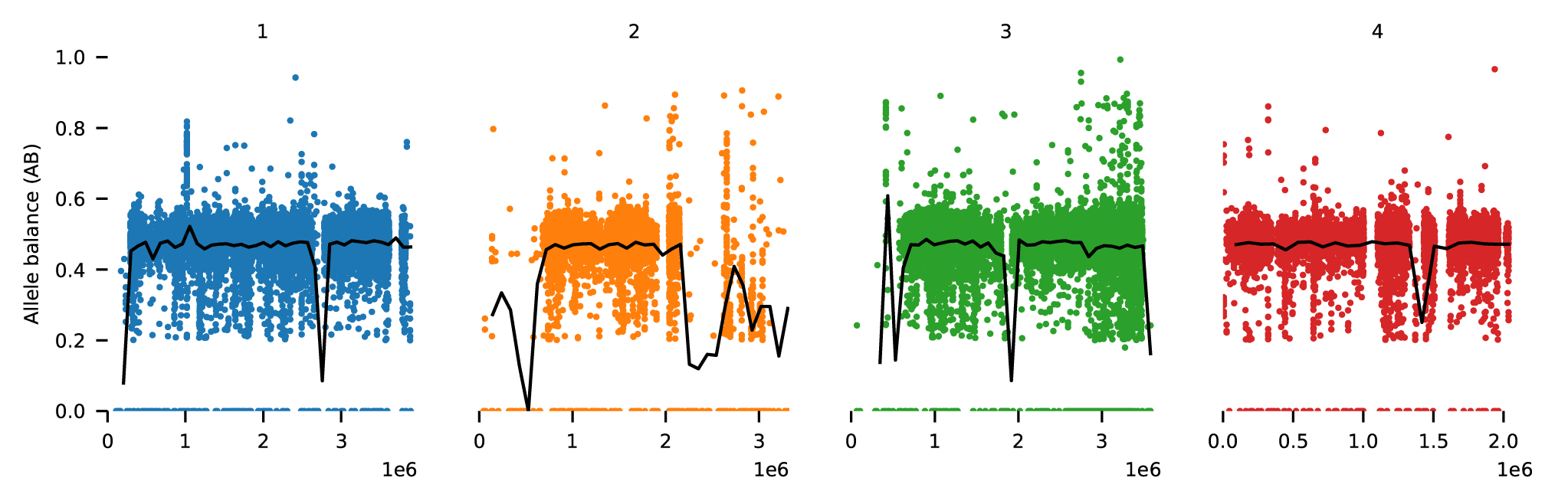


Figure S1. Haplotype sampling of CBS 11270. Alignment of paired-end Illumina reads to chromosome sequences of *Brettanomyces bruxellensis* CBS 11270 was performed using BWA version 0.7.4 and analyzed with FreeBayes version 1.1.0 for variant calling. The Allele Balance^[[1]](#endnote-1)^ (AB) values resulting from variant analysis were used as Y axis coordinates and corresponding position on chromosomes as X axis coordinates.

1. Allele balance at heterozygous sites: a number between 0 and 1 representing the ratio of reads showing the reference allele to all reads, considering only reads from individuals called as heterozygous [↑](#endnote-ref-1)
