# Supplementary material for: Chromosomal genome assembly of the ethanol production strain CBS 11270 indicates a highly dynamic genome structure in the yeast species Brettanomyces bruxellensis
Source: PLoS One. 2019 May 1;14(5):e0215077. doi: 10.1371/journal.pone.0215077 (PMC6493715; doi:10.1371/journal.pone.0215077)
Supplement: S2 Fig — (DOCX) [file pone.0215077.s018.docx]

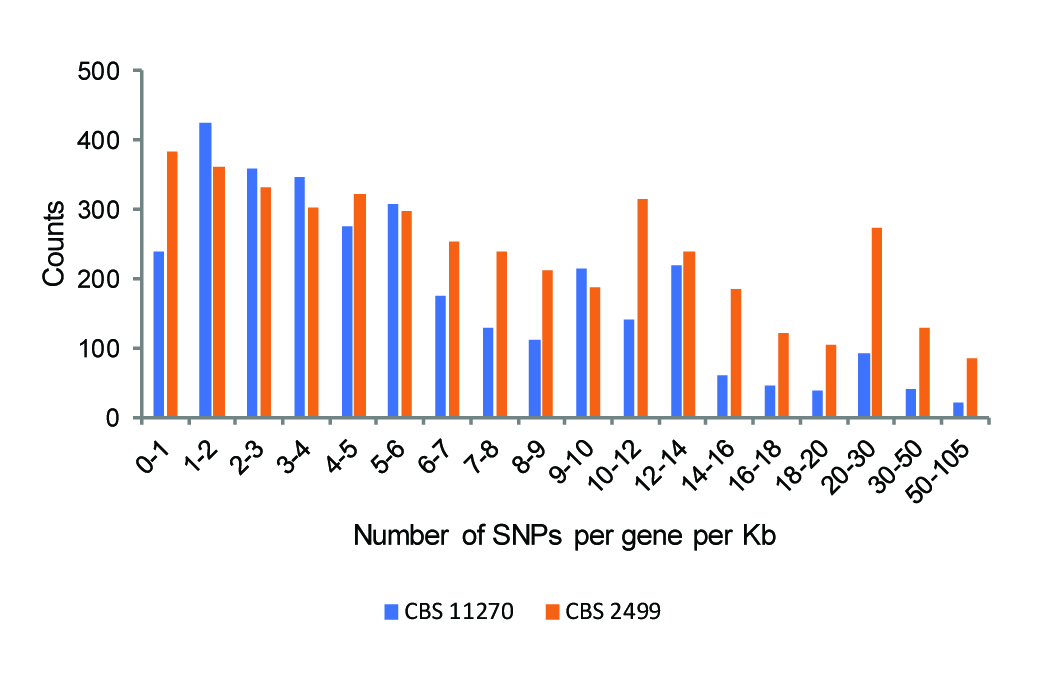


S2 Figure. Distribution of SNPs per Kbp gene in the genome of *B. bruxellensis* CBS 11270 (blue) and CBS 2499 (red). Alignment of paired-end Illumina reads generated from sequencing of *B. bruxellensis* CBS 11270 genome was performed using BWA version 0.7.4 and SNPs were identified using GATK HaplotypeCaller version 2.8-1.

58 genes of *B. bruxellensis* CBS 2249 with number of SNPs per Kbp per gene higher than 105 were not shown. The high value of SNPs per Kbp per gene (between 105 and 2909) arose from short length of genes annotated in CBS 2249.
