# Supplementary material for: Chromosomal genome assembly of the ethanol production strain CBS 11270 indicates a highly dynamic genome structure in the yeast species Brettanomyces bruxellensis
Source: PLoS One. 2019 May 1;14(5):e0215077. doi: 10.1371/journal.pone.0215077 (PMC6493715; doi:10.1371/journal.pone.0215077)
Supplement: S3 Fig — (DOCX) [file pone.0215077.s019.docx]

A.


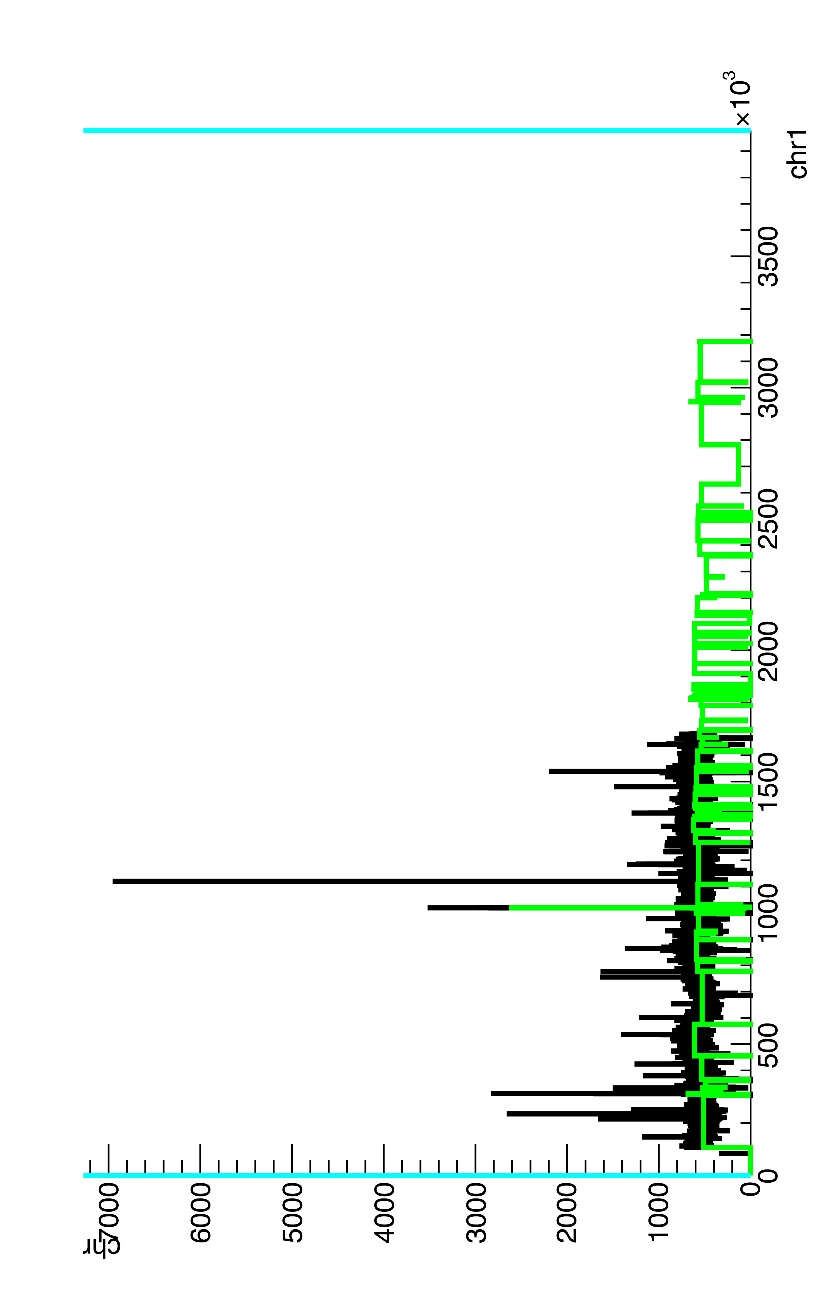


B.


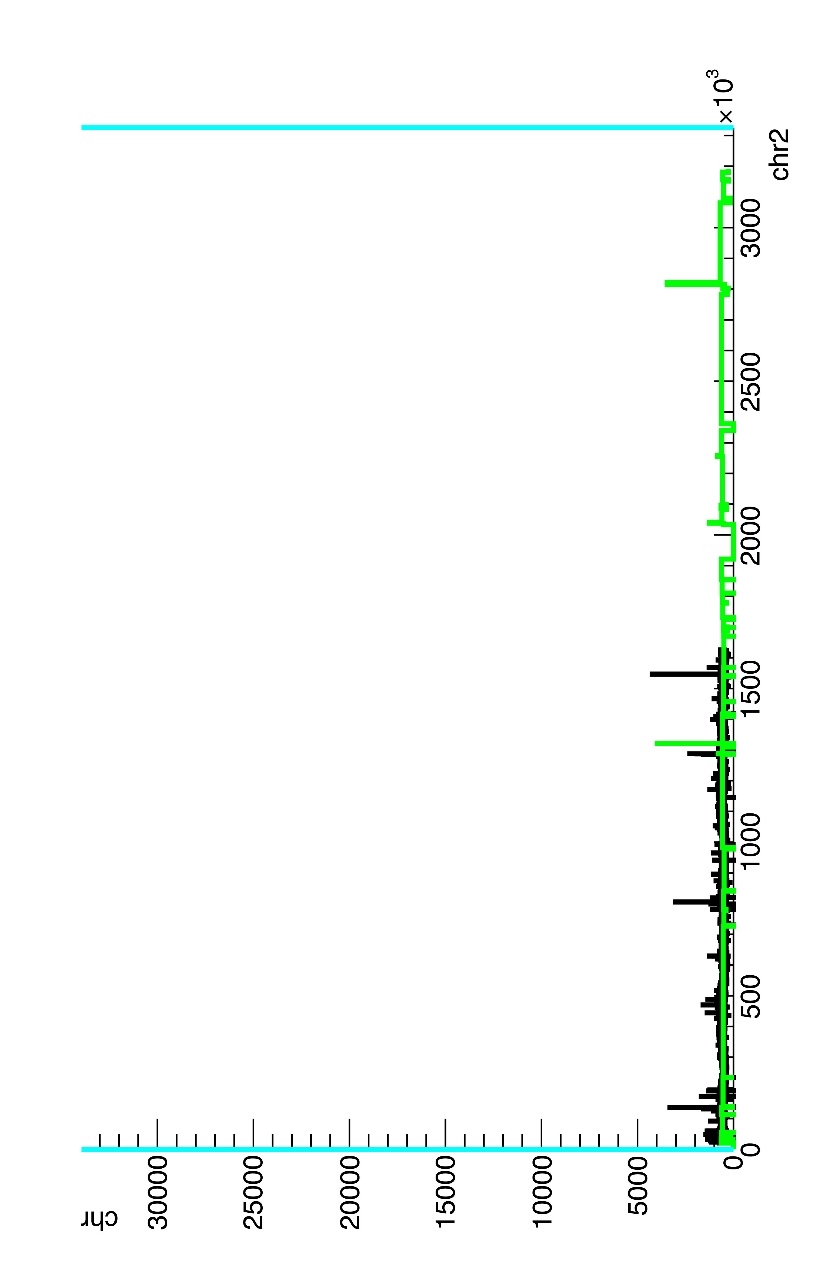


C.


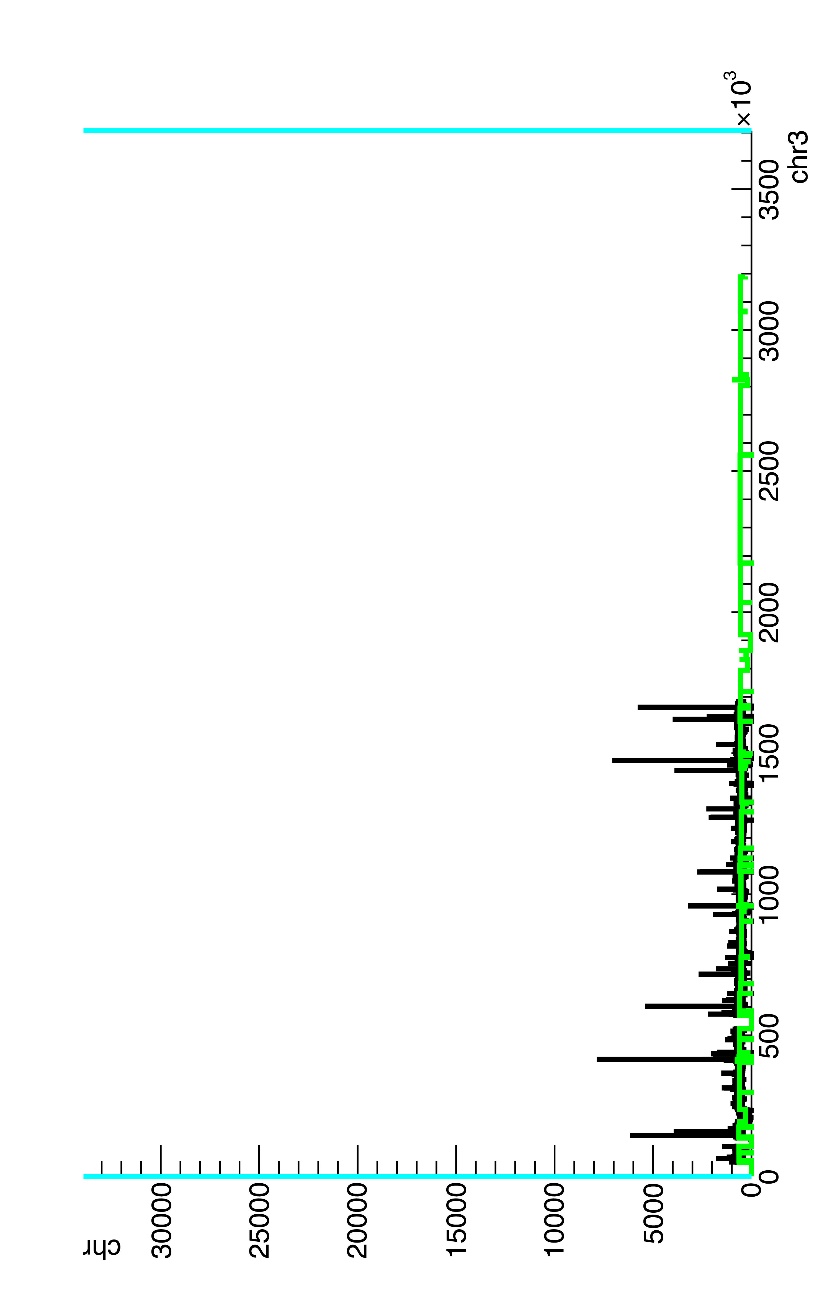


D.


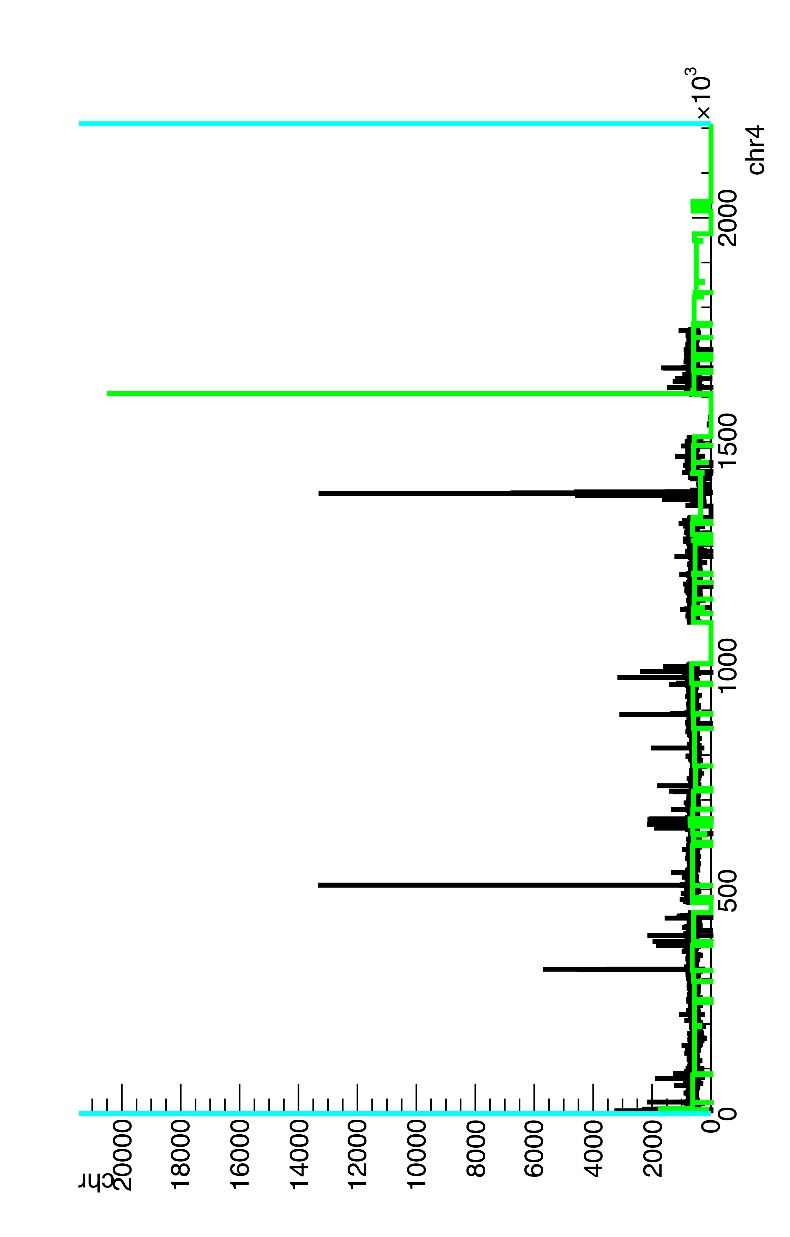


S3 Figure. Variation in coverage of chromosomes in *Brettanomyces bruxellensis* CBS 11270.

Alignment of paired-end Illumina reads to chromosome sequences of *B. bruxellensis* CBS 11270 was performed using BWA version 0.7.4 and copy number variation was identified using CNV tool version 0.3. in CBS 11270 chromosome 1 (A); chromosome 2 (B); chromosome 3 (C); and chromosome 4 (D). Y axis shows the read depth (copy number) and X axis shows the position on chromosome.
