# Supplementary material for: Chromosomal genome assembly of the ethanol production strain CBS 11270 indicates a highly dynamic genome structure in the yeast species Brettanomyces bruxellensis
Source: PLoS One. 2019 May 1;14(5):e0215077. doi: 10.1371/journal.pone.0215077 (PMC6493715; doi:10.1371/journal.pone.0215077)
Supplement: S4 Fig — (DOCX) [file pone.0215077.s020.docx]

A.


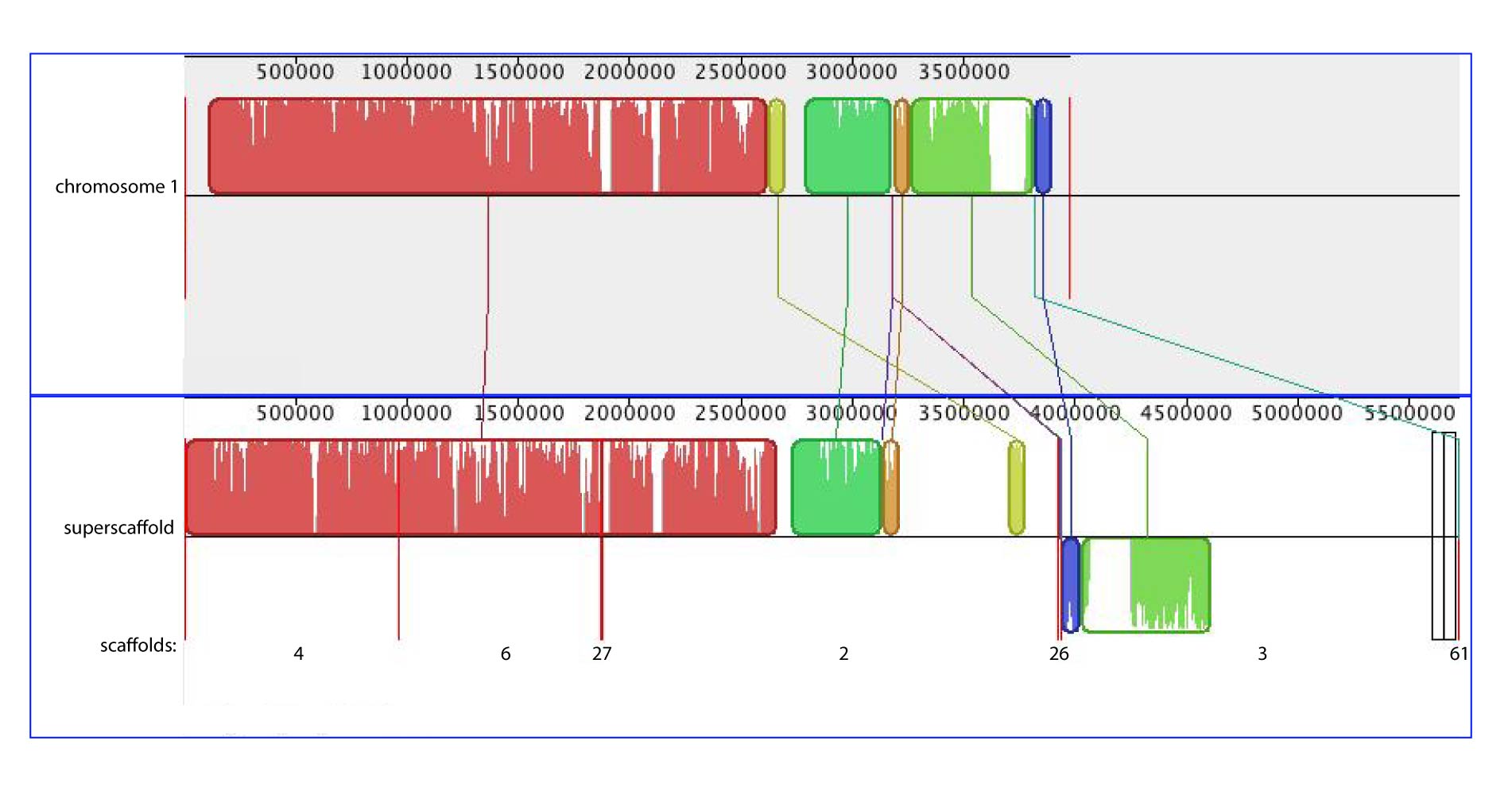


B.


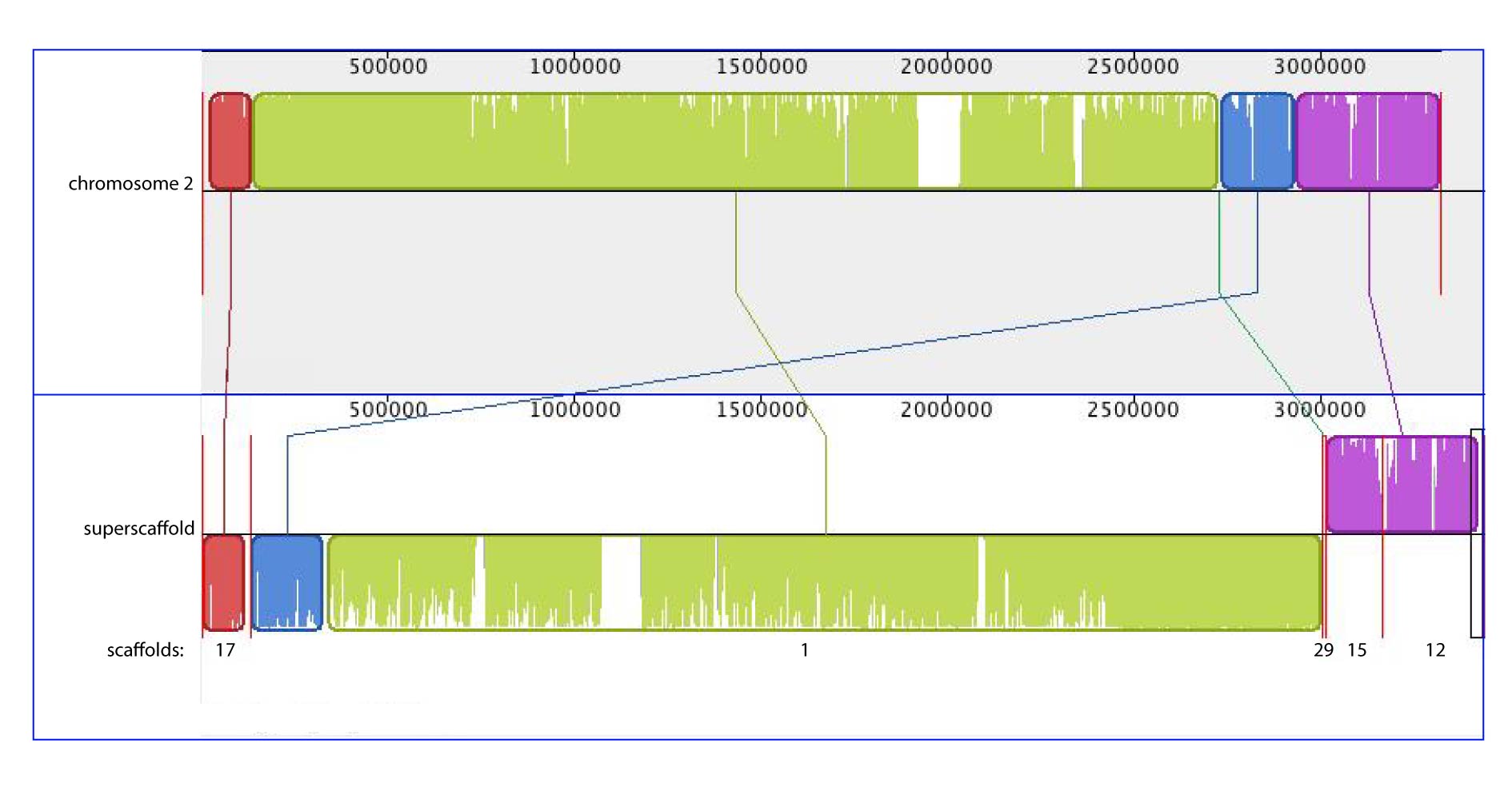


C.


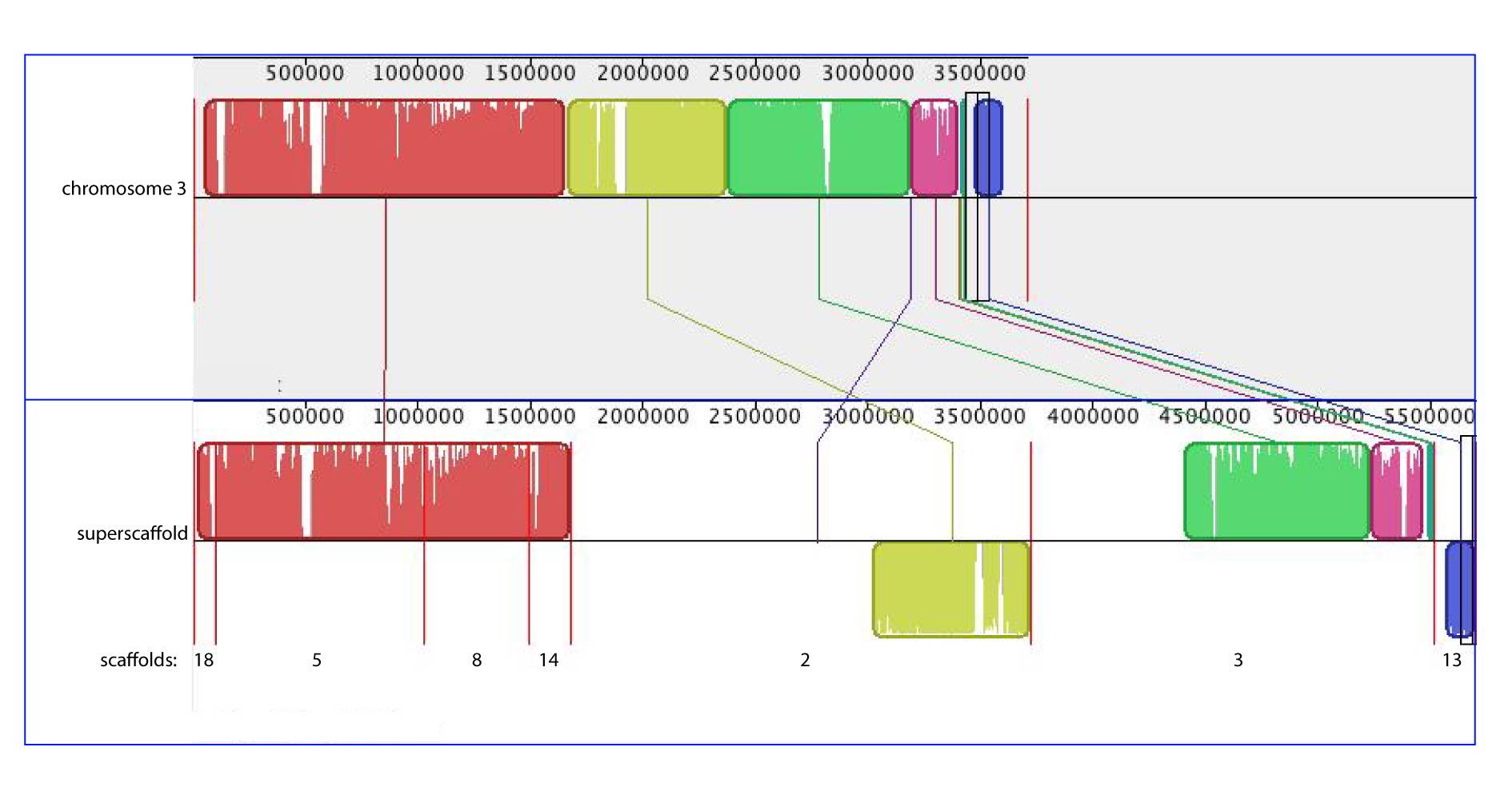
D.


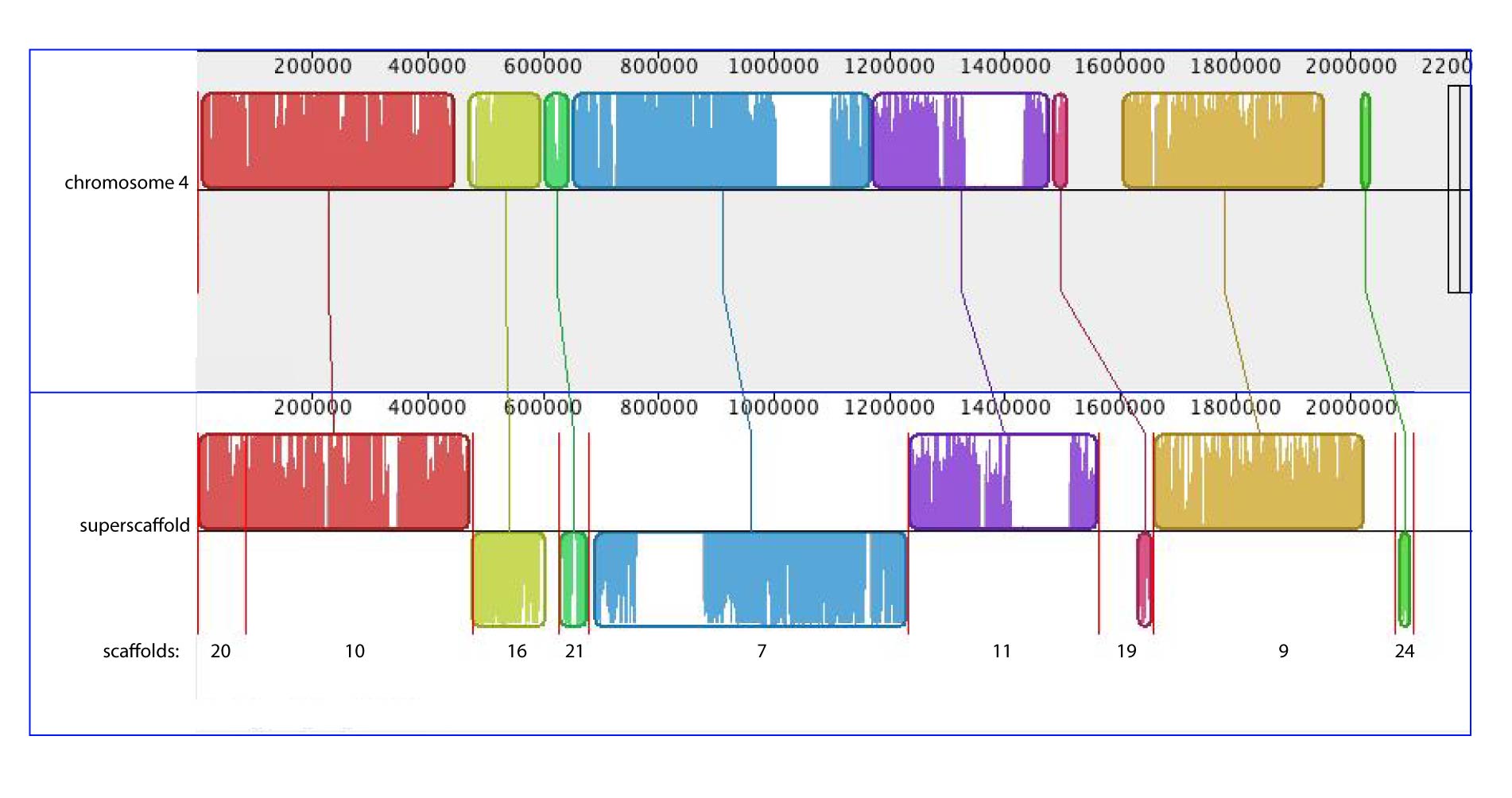


Figure S4. Alignment of genomes *B. bruxellensis* CBS 11270 and CBS 2499, using Mauve alignment Viewer. CBS 2499 scaffolds were aligned against CBS 11270 chromosome 1 (A); chromosome 2 (B); chromosome 3 (C); and chromosome 4 (D). The alignment display shows one horizontal "panel" per input DNA sequence. Each panel contains the name of the DNA sequence, a scale showing the sequence coordinates for that fragment, and a black horizontal line. A colored block indicates a fragment of the genome sequence that aligned to a part of the genome of the other strain, and is presumably homologous. When a block lies above the black horizontal line the aligned region is in the forward orientation relative to the CBS 11270 genome sequence. Blocks below the black horizontal line show regions that align in the reverse complement (inverse) orientation. Regions outside colored blocks are non- homologous to the input sequence. Inside each block Mauve displays a similarity profile of the genome sequence. Regions that are completely white do not have homology to input DNA sequence.
